# Lipopolysaccharide derived from the digestive tract activates inflammatory gene expression and inhibits casein synthesis in the mammary glands of lactating dairy cows

**DOI:** 10.18632/oncotarget.7371

**Published:** 2016-02-14

**Authors:** Kai Zhang, Guangjun Chang, Tianle Xu, Lei Xu, Junfei Guo, Di Jin, Xiangzhen Shen

**Affiliations:** ^1^ College of Veterinary Medicine, Nanjing Agricultural University, Nanjing, P. R. China

**Keywords:** lipopolysaccharide, signaling pathway, inflammatory response, casein, mammary gland, Immunology and Microbiology Section, Immune response, Immunity

## Abstract

To meet the nutrition requirements of lactation, dairy cows are usually fed a high concentrate diet (HC). However, high-grain feeding causes subacute ruminal acidosis (SARA), a metabolic disorder that causes milk protein depression. This study aimed to investigate the effect of lipopolysaccharide (LPS) released in the rumen on inflammatory gene expression and casein synthesis in mammary glands of lactating dairy cows fed a HC diet. We found that milk protein was significantly decreased in the HC group after 15 weeks of feeding. Overall, LPS concentrations in the rumen fluid, lacteal artery and vein were increased in the HC group. Transcriptome microarray was used to evaluate alterations in the signaling pathway in mammary glands. Signaling pathways involved in inflammatory responses were activated, whereas those involved in protein synthesis were inhibited in the HC group. mRNA expression involved in inflammatory responses, including that of TLR4, NF-кB and pro-inflammatory genes, was increased in the HC group, while αs1-casein (CSN1S1), β-casein (CSN2), mTOR and S6K gene expression were decreased. Moreover, protein expression was consistent with the corresponding gene expression. After feeding with an HC diet, LPS derived from the rumen increased inflammatory gene expression and inhibited casein synthesis in the mammary glands of lactating dairy cows fed a HC diet.

## INTRODUCTION

Dairy cows are often fed a high concentrate diet (HC) to meet the nutritional demands of lactation. However, the long-term overfeeding of cattle with a diet rich in concentrate results in a metabolic disorder termed subacute ruminal acidosis (SARA), in which the rumen pH falls below 5.6 for more than 3 hours per day [1]. One consequence of SARA is the depression of milk quality and quantity [2, 3], and previous studies indicated that SARA reduced milk protein production [4, 5]. The rumen contains gram-negative bacteria, of which lipopolysaccharide (LPS) is an important cell wall component. Feeding cattle with HC decreases the rumen pH level, causing gram-negative bacteria to be lysed in the rumen and releasing free endogenous LPS [6]. LPS can then be translocated into the bloodstream from the digestive tract [7] and enter the mammary gland, initiating inflammatory responses that result in reduced productivity of the animal [8, 9].

LPS is a strong activator of innate immune responses and is recognized by TLR4, which then activates the immune response [10]. The TLR4 recognition of LPS is facilitated by the accessory molecule LPS-binding protein (LBP) and cluster of differentiation antigen 14 (CD14) [11, 12]. NF-кB is the key transcription factor that regulates the expression of cytokine genes. Normally, NF-кB is located in the cytoplasm in the inactive form combined with inhibitory кB. It is activated by phosphorylation and released from the NF-кB-IкB compound; it then enters the nucleus. In nucleus, NF-кB connects with the promoter and regulates the transcription of TNF-α and IL-1β, IL-6, and IL-8 [13].

Protein is the most important nutrient in milk, and the level of milk protein is a crucial parameter of milk quality. Casein accounts for 80% of milk protein in dairy cows and is composed of four major groups: αS1-casein, αS2-casein, β-casein, and κ-casein [14], of which αS1-casein is the most abundantly expressed milk protein in dairy cows. The main regulator of milk protein expression in the bovine mammary gland is the Janus kinase (JAK) signal transducer and activator of transcription (STAT) signaling pathway and mTOR signaling pathway[15, 16]. A recent study in ruminants highlighted a role for mTOR in the regulation of milk protein synthesis [17-19]. It was reported that experimentally induced mastitis in dairy cows that were challenged with an intra-mammary infusion containing *E. coli* had reduced casein synthesis and αS1-casein mRNA expression[20]. Some studies have indicated that CpG (cytosine-phosphate-guanine) dinucleotide methylation on the promoter of the αS1-casein gene and chromatin remodeling depressed αS1-casein synthesis, which revealed that the reason for the prohibited synthesis of αS1-casein was translational but not transcriptional regulation [21]. Another study demonstrated that exogenous LPS injection into the mammary glands of dairy cows induced immune responses and reduced casein synthesis [22, 23]. Global transcriptome microarray assay showed that LPS priming enhanced the expression of immune effect or molecules in mammary epithelia cells from cows [24, 25]. To date, most studies have focused on the association of immune responses and casein synthesis in mammary glands of dairy cows stimulated by exogenous LPS stimulation. However, little is known about the relationship between inflammatory responses and casein synthesis in the mammary glands of dairy cows challenged by endogenous LPS from the rumen. Therefore, the present study was conducted to investigate the effects of endogenous LPS released in the rumen on inflammatory responses and casein synthesis in the mammary glands of lactating dairy cows fed HC.

## RESULTS

### Milk protein and SCC

**Figure 1:**
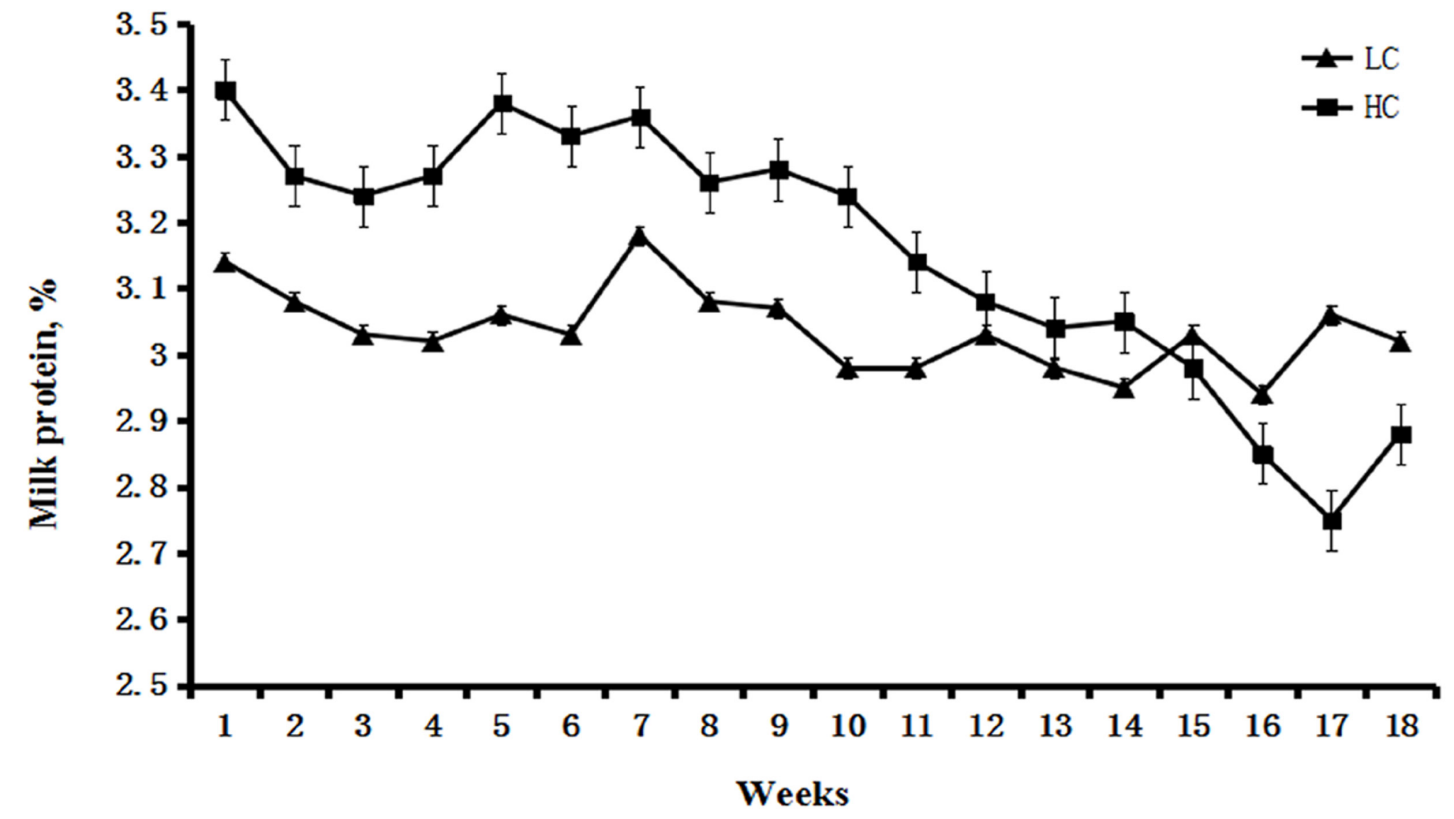
Percentage of milk protein in dairy cows fed low concentrate (LC) or high concentrate (HC) diets Percentage of milk protein in the HC group was higher than that in the LC group between weeks 1 and 15 after the initiation of the diet. However, after 15 weeks, the percentage of milk protein was lower in the HC group compared with the LC group.

**Figure 2:**
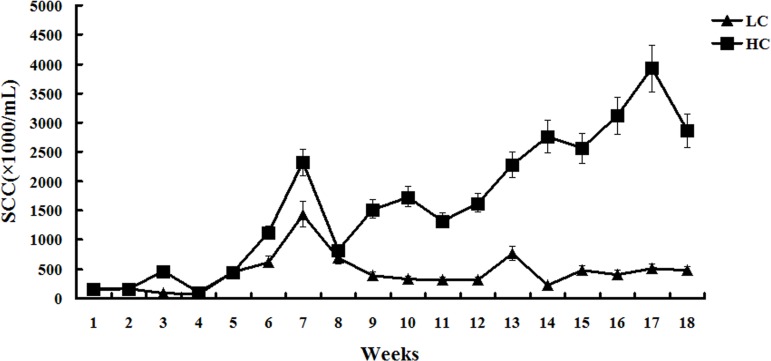
Somatic cell count of milk in dairy cows fed low concentrate (LC) or high concentrate (HC) diets At 8 weeks after the initiation of the experiment, the somatic cell count of milk was similar between the HC and LC groups. After 8 weeks of diet, the somatic cell count of milk in the LC group remained steady, but was abruptly increased in the HC group.

The percentage of milk protein was higher in the HC group compared with the LC group between weeks 1 to 15. However, from week 15, the percentage of milk protein significantly decreased in the HC group compared with the LC group (Figure 1). Milk SCC was similar between the HC and LC groups before week 8. From week 9 to 18, SCC remained steady in the LC group, but was significantly increased in the HC group (*P* < 0.01, Figure 2).

### Rumen pH, LPS content and primary pro-inflammatory cytokines

The mean rumen pH was lower when cows were fed HC compared with LC (*P* < 0.05, Table 3). The mean pH value in the HC group was lower than 5.6 for more than 3 hours per day, which indicated that SARA was experimentally induced by HC.

**Table 1:** Ingredients, nutrient composition and forage to concentrate ratio (F:C) of experimental diets

Ingredient, % DM	Percentage (%) of ingredients in different diets (air dry matter)
	HC	LC
Corn silage	30	20
Alfalfa hay	30	20
Corn	24.3	32
Bran	0	12.4
Soybean meal	13.5	13
Calcium phosphate dibasic	0.85	0.45
Powder	0	0.8
Salt	0.35	0.35
Premix*	1	1
F:C	4:6	6:4
Nutrient composition		
NE,MJ/kg	6.36	6.71
CP %	16.99	16.92
EE %	3.93	4.07
NDF %	36.54	31.45
ADF %	22.51	17.56
NFC %	33.76	39.32

* Premix contained 5.25 g/kg of Fe, 1.2 g/kg of Cu, 5.5 g/kg of Mn, 13 g/kg of Zn, 50 mg/kg of Co, 27 mg/kg of Se, 170 mg/kg of I, 1,900 ku/kg of vitamin A, 250 ku/kg of vitamin D, and 3 g/kg of vitamin E.

**Table 2:** The primer sequences of target and internal reference genes used in qRT-PCR

Gene	Forward primer	Reverse primer	Genebank accession	PCR products (bp)
TLR4	GGACCCTTGCGTACAGGTTG	GGAAGCTGGAGAAGTTATGGC	NM_174198.6	244
LBP	GCAAGATCACTGGATTCTTGGA	AAAACAGGAAGTCCTTGTGGATC	NM_001038674.2	228
IL1b	GGCCAAAGTCCCTGACCTCT	CTGCCACCATCACCACATTC	NM_174093.1	167
IL6	GGAGGAAAAGGACGGATGCT	GGTCAGTGTTTGTGGCTGGA	NM_173923.2	227
IL-8	CCTCTTGTTCAATATGACTTCCA	GGCCCACTCAATAACTCTC	NM_173925.2	170
TNF-α	CTTCTGCCTGCTGCACTTCG	GAGTTGATGTCGGCTACAACG	AF_348421.1	156
NF-κB	ATACGTCGGCCGTGTCTAT	GGAACTGTGATCCGTGTAG	NM_001045868.1	129
CSN1S1	CTTTTCAGACAATTCTACCAGCT	AATTCACTTGACTCCTCACCAC	NM_181029.2	170
CSN2	AGTCCAAAGTCCTGCCTGTTCC	TGCCATATTTCCAGTCGCAGTC	NM_181008.2	193
JAK2	ACAGGGGCTGGCGTTCA	TATTGGTAACCAACAGCTCAAGG	XM_002689603.1	146
mTOR	ATGCTGTCCCTGGTCCTTATG	GGGTCAGAGAGTGGCCTTCAA	XM_001788228.1	199
STAT5	AAGACCCAGACCAAGTTCGC	AGCACCGTGGCAGTAGCAT	NM_001012673.1	203
4EBP1	GGCAGGCGGTGAAGAGTC	CCTGGGCTGCGGGAT	BC120290.1	177
S6K	GGACATGGCAGGGGTGTTT	GGTATTTGCTCCTGTTACTTTTCG	NM_205816.1	162
GAPDH	GGGTCATCATCTCTGCACCT	GGTCATAAGTCCCTCCACGA	XM_001252479	177

**Table 3:** Rumen pH, LPS and primary pro-inflammatory cytokine content in the rumen and plasma in dairy cows fed low concentrate (LC) or high concentrate (HC) diets

Item	LC	HC	SEM	*P*-Value
Mean pH value	6.02	5.90	0.03	<0.05
Time<pH5.6, h/d	1.65	3.72	0.50	<0.01
LPS in rumen, kEU/mL	47.2	79	7.9	<0.01
LPS in plasma of lacteal artery, EU/mL	0.47	0.86	0.06	<0.01
LPS in plasma of lacteal vein, EU/mL	0.12	0.27	0.03	<0.01
in plasma of lacteal vein, ng/mL	0.39	0.95	0.07	<0.05
IL-6 in plasma of lacteal vein, pg/mL	108.42	564.32	10.17	<0.01
TNF-α in plasma of lacteal vein, fmol/mL	10.86	15.68	0.38	<0.05

LPS content in the rumen fluid of the HC group was significantly increased compared with the LC group 4 h after feeding (*P* < 0.01). The LPS concentration in plasma from the lacteal artery was 0.47 EU/mL in the LC group and was significantly higher in the HC group, 0.86 EU/mL (*P* < 0.01). LPS content in plasma from the lacteal vein of the HC group was significantly elevated (from 0.12 EU/mL to 0.27 EU/mL) compared with the LC group (*P* < 0.01, Table 3). Thus, higher LPS levels were translocated into mammary glands from the bloodstream in the HC group.

The plasma concentrations of primary pro-inflammatory cytokines IL-1β (*P* < 0.01), IL-6 (*P* < 0.05) and TNFα (*P* < 0.01) in the lacteal vein were significantly increased in the HC group compared to the control group (Table3).

### Alteration of signaling pathways

**Figure 3:**
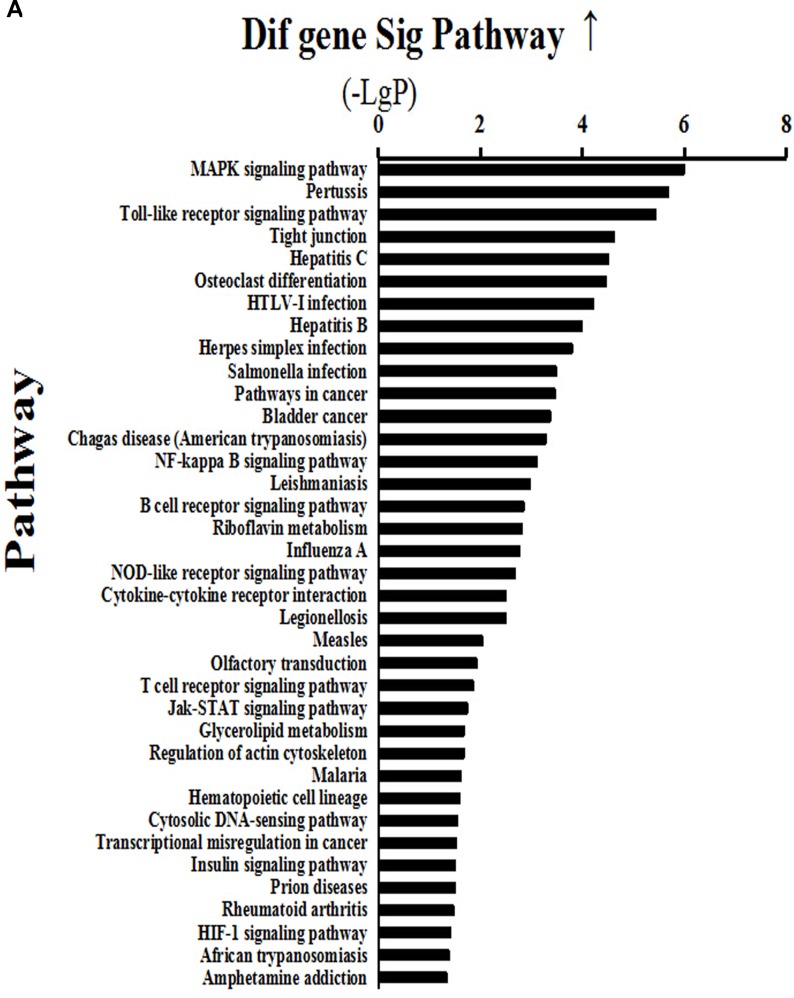

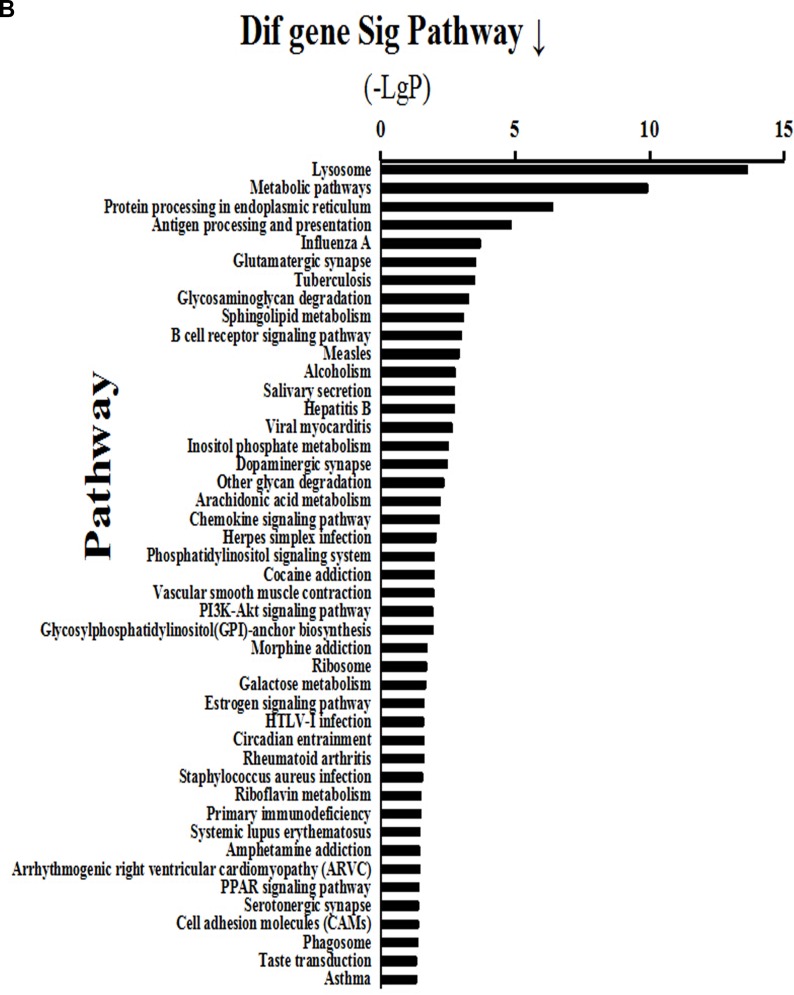
Activated (a) and depressed (b) signaling pathways in mammary glands of dairy cows fed a high concentrate (HC) diet **A.** Thirty-seven signaling pathways were activated >2-fold in the HC group compared with the LC group. Among them, MAPK, Toll-like receptor (TLR) and NF-κB signaling pathways were associated with inflammatory responses. **B.** Forty-five signaling pathways were inhibited >2-fold in the HC group compared with the LC group, of which protein processing in endoplasmic reticulum and PI3K-AKT1-mTOR signaling pathways were involved in protein synthesis.

Analysis of signaling pathways was performed according to gene expression profiling by the transcriptome microarray of mammary gland tissues. The results showed that 37 signaling pathways were activated above two-fold in the HC group compared with the LC group (Figure 3A). Activated major signaling pathways associated with inflammatory responses were the MAPK, TLR and NF-κB signaling pathways. Of these, the MAPK signaling pathway was activated 6-fold, the TLR pathway 5-fold, and the NF-κB pathway >3-fold. In addition, the results showed that 45 signaling pathways were inhibited >2-fold in the HC group compared with the LC group (Figure 3B), of which protein processing in the endoplasmic reticulum and the PI3K-AKT1-mTOR signaling pathway were involved in protein synthesis. Protein processing in the endoplasmic reticulum was reduced 10-fold, while the PI3K-AKT1-mTOR signaling pathway was inhibited 3-fold.

### mRNA expression of inflammatory response genes

**Figure 4:**
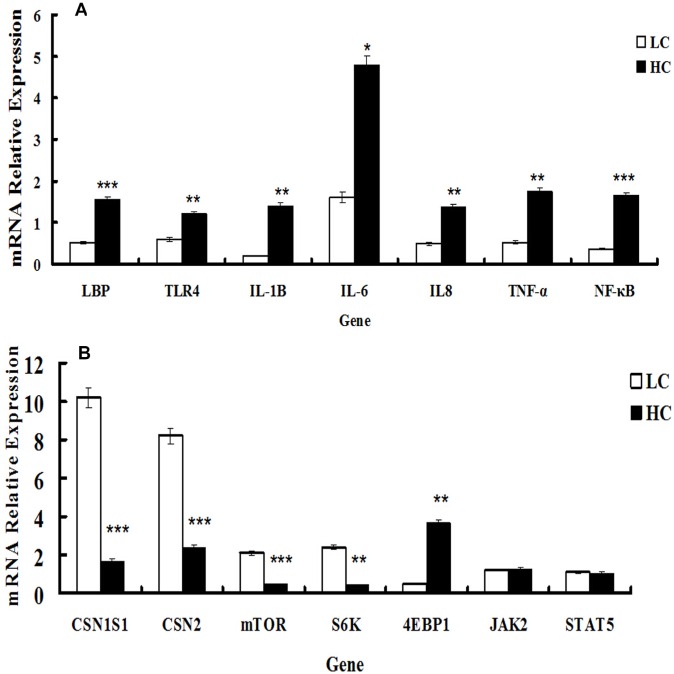
mRNA expression of genes involved in inflammatory responses and casein synthesis in mammary glands of dairy cows fed low concentrate (LC) or high concentrate (HC) diets **A.** mRNA expression of genes responsible for inflammatory responses (TLR4, LBP, IL-1β, IL-6, IL-8, NF-κB and TNF-α) was determined. All genes were up-regulated significantly in dairy cows fed a high concentrate (HC) diet. Significant differences are indicated (*P < 0.1, **P < 0.05, ***P < 0.01, paired *t*-test). **B.** Gene expression of casein and mTOR signaling pathway-related genes (CSN1S1, CSN2, mTOR and S6K) were down-regulated in dairy cows fed a high concentrate (HC) diet, but the expression of 4EBP1 was elevated. The expression of JAK2 and STAT5 was not significantly different between the HC and low concentrate (LC) groups. Significant differences are indicated (**P < 0.05, ***P < 0.01, paired *t*-test).

qRT-PCR analysis was performed to determine the relationship between alterations in signaling pathways associated with inflammatory responses in the mammary gland and the transcriptional levels of seven genes involved in inflammatory responses in the HC and LC groups. mRNA expression of LBP and TLR4 was significantly up-regulated in the HC group compared with the LC group (*P* < 0.01 and *P* < 0.05, respectively). The transcription factor NF-κB, which is implicated in the regulation of the inflammatory response, showed a>2-fold difference in mRNA expression between the HC group and LC group. The expression of cytokine mRNA for IL-1β, IL-8, and TNF-α was significantly increased in the HC group compared with the LC group (*P* < 0.05), whereas no significant difference was observed for mRNA expression of IL-6 due to the high variability between individual values (Figure 4A). These results were consistent with the changes in signaling pathway analysis.

### mRNA expression of genes associated with casein

The expression of genes involved in protein synthesis was altered by HC feeding. The mRNA expression of both CSN1S1 and CSN2 was significantly down-regulated in the HC group compared with the LC group (*P* < 0.01). The expression of mTOR and S6K mRNA levels involved in the mTOR signaling pathway were remarkably decreased, whereas 4EBP1 mRNA expression was markedly elevated (*P* < 0.05) in the HC group compared with the LC group. The expression of the JAK2 and STAT5 genes was not significantly different between the HC and LC groups (Figure 4B).

### Protein expression of TLR4 and mTOR signaling pathway

**Figure 5:**
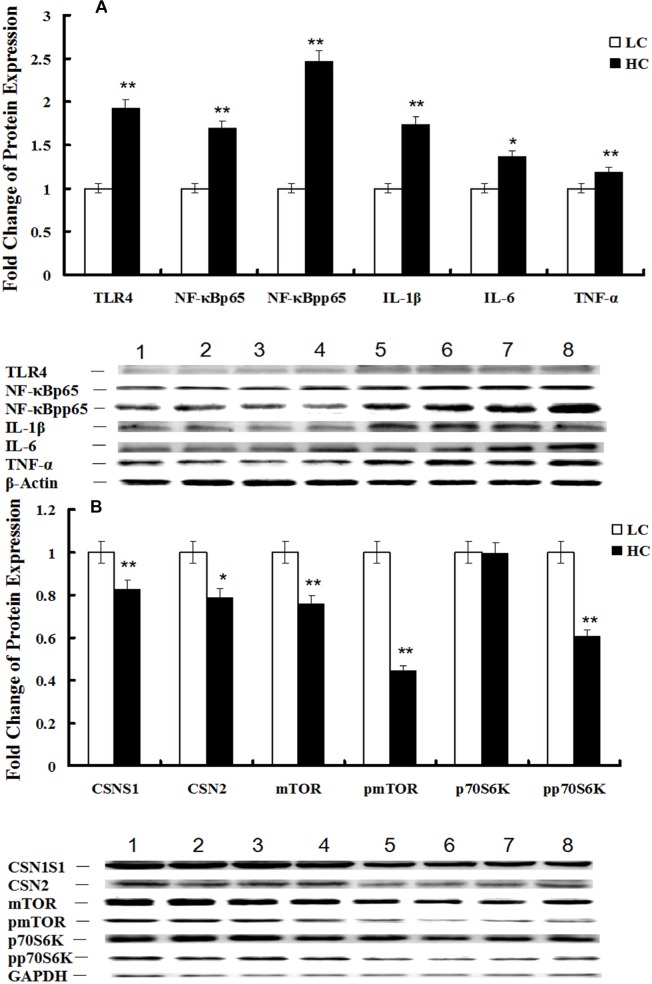
Protein expression of the TLR4 and mTOR signaling pathways in mammary glands of dairy cows fed low concentrate (LC) or high concentrate (HC) diets **A.** Protein expression of TLR4, NF-κBp65, NF-κBpp65, IL-1β, IL-6 and TNFα in mammary glands of dairy cows fed HC and LC diets was determined by western blotting. The protein was quantified by band density, and β-actin was used as an internal reference. Bands 1-4 represent LC group samples and bands 5-8 represent HC group samples (*P < 0.10 and **P < 0.05). **B.** Protein expression of CSN1S1, CSN2, mTOR, pmTOR, p70S6K and pp70S6K in mammary glands of dairy cows fed HC and LC diets was determined by western blotting. The protein was quantified by band density, and GAPDH was used as an internal reference. Bands 1-4 represent LC group samples and bands 5-8 represent HC group samples (*P < 0.10 and **P < 0.05).

Western blotting of the TLR4 (*P* < 0.01), NF-κBp65 (*P* < 0.05), NF-κBpp65 (*P* < 0.1), IL-1β (*P* < 0.01), IL-6 (*P* < 0.05) and TNFα (*P* < 0.01) proteins in mammary glands demonstrated that the NF-κB signaling pathway was activated in the HC group (Figure 5A), while the expression of the CSN1S1 (*P* < 0.1), CSN2 (*P* < 0.1), mTOR (*P* < 0.01), pmTOR (*P* < 0.01) and pp70S6K (*P* < 0.01) proteins in mammary glands were decreased in the HC group compared with the LC group (Figure 5B). Changes in protein levels were consistent with the corresponding gene expression.

## DISCUSSION

The results of the present study demonstrated that endogenous LPS released in the rumen can increase inflammatory gene expression and inhibit casein synthesis in mammary glands of lactating dairy cows fed HC. These findings provide insight into the role of endogenous LPS on the performance of dairy cows offered HC and the relationship between lactation and immune defense.

Dairy cows are often fed a relatively high proportion of grains or easily fermentable carbohydrates in the diet to promote high milk yields, which cause the accumulation of volatile fatty acid (VFA) and lactic acid in the rumen, and lead to SARA due to rapidly decreased rumen pH [26, 27]. In the present study, dairy cows fed HC for 18 weeks exhibited a significantly lower rumen pH, which met the criterion for SARA diagnosis, indicating that SARA was experimentally induced by HC.

It is thought that SARA causes milk protein depression. Inconsistent responses to milk protein in experimentally induced SARA are related to the duration of SARA, with short periods being more likely to have no effect on milk protein content. Our long-term study demonstrated that the percentage of milk protein decreased significantly at 15 weeks after the initiation of HC, whereas the percentage of milk protein remained steady in the LC group. Milk SCC is a very important indicator of mammary gland status and milk quality [28]. The present study demonstrated that milk SCC in the HC group was much higher in the late stage of the experiment compared with the LC group and that milk protein in the HC group was reduced.

During experimentally induced SARA, rumen pH values decrease rapidly, causing a large number of Gram-negative bacteria in the rumen to lyse, subsequently releasing free endogenous LPS into the rumen. The current study showed that LPS content in the rumen and plasma from the lacteal artery and vein of dairy cows was significantly increased in the HC group. Therefore, higher levels of LPS were translocated into the mammary gland in the HC group.

LPS-induced TLR4 activation is mainly mediated by the adaptor molecule myeloid differentiation primary-response gene 88 (MyD88) [29]. Then, the transduced signal activates downstream signaling molecules, including NF-κB, mitogen-activated protein kinases (MAPKs), and TRIF-IFN-regulatory factor (IRF3) [30, 31]. In addition, the JAK2-STAT5 pathway is activated by LPS/TLR4, although the adaptor molecule for JAK2 has not yet been identified [32]. The activation of these signaling cascades leads to the abundant secretion of the proinflammatory cytokines TNF-α, IL-1β and IL-6 [33, 34].. The expression of proinflammatory mediators is primarily modulated by the NF-κB and MAPK pathways. Most inflammatory genes contain NF-κB binding sites within their promoters and therefore depend partly on NF-κB for their expression [35, 36].

The signaling pathway analysis in our study was based on a transcriptome microarray and showed that the TLR, NF-κB, MAPK and JAK-STAT signaling pathways were activated, while protein processing in the endoplasmic reticulum and the phosphatidylinositol 3-kinase (PI3K)-AKT1-mTOR signaling pathway, which is closely related with casein synthesis, were depressed in the HC group. Although the JAK-STAT signaling pathway might be an essential component for the proper expression of milk protein genes in non-ruminants [37], its role in regulating milk protein expression seems to be weak in bovine populations [38]. Our results revealed that the translocation of endogenous LPS into the mammary gland enhanced the expression of inflammatory genes, whereas the expression of genes associated with casein synthesis was inhibited.

A recent study in ruminants emphasized the action of the mTOR signaling pathway in regulating milk protein synthesis. The Akt/mTOR signaling pathways play important roles on phosphorylation of protein translational regulators S6K and 4EBP1 [39, 40]. It is reported that eukaryotic translation initiation factor (eIF-4F) performs an important function in stimulating protein translation, and the role of eIF-4E can be inhibited by the phosphorylation of 4EBP1; thus, the translation of proteins is prohibited [41]. Previously, mTOR was shown to be inhibited in mice or humans after LPS stimulation [42]. The inhibitory effects of endogenous LPS on milk synthesis in dairy cows were assessed in the current study. The mRNA expression of genes responsible for the inflammatory response (TLR4, LBP, IL-1β, IL-6, IL-8, NF-κB and TNF-α) was significantly increased, consistent with the results of exogenous LPS-induced mastitis by infusion [43]. The protein expression of TLR4, NF-κBp65, NF-κBpp65, IL-1β, IL-6 and TNFα was increased in the HC group. This showed that the TLR4 signaling pathway in the HC group was activated. Gene expression of casein and signaling pathway-related genes (CSN1S1, CSN2, mTOR, and S6K) were decreased, except for 4EBP1 in the HC group. Increased 4EBP1 gene expression agreed with the negative role of 4EBP1 in protein translation [47]. Treatment with HC had no obvious effect on JAK2 and STAT5 gene expression. In the current study, the protein expression of CSN1S1, CSN2, mTOR, pmTOR and pp70S6K was decreased, consistent with the mRNA expression of their corresponding genes. This indicated that endogenous LPS from long-term HC feeding inhibited the synthesis of CSN1S1 and CSN2 in mammary glands. However, the correlation between the inflammatory response and casein synthesis requires further study.

In conclusion, long-term feeding of HC to lactating cows causes milk protein depression. The inflammatory response in mammary glands induced by LPS derived from the rumen is associated with reduced casein synthesis. Future research is required to elucidate the molecular mechanisms involved. Maintaining a healthy rumen in dairy cows to decrease the release of LPS in the rumen is a strategy to improve the percentage of milk protein.

## MATERIALS AND METHODS

### Ethics statement

The experimental protocol was approved by the Animal Care Committee of Nanjing Agricultural University in accordance with the Guidelines for Experimental Animals of the Ministry of Science and Technology (2006, Beijing, China).

### Animals, diet, and experimental design

Twelve lactating Holstein cows (455 ± 28 kg live weight) fitted with a rumen fistula were randomly divided into two groups. One group was fed HC comprised of 40% forage and 60% concentrate as a treatment, and the other group received a low concentrate diet (LC) comprised of 60% forage and 40% concentrate as a control over a 20-week experimental period. The ingredients and nutritional composition of the diets are presented (Table 1). Cows were fed at 04:00, 12:00, and 20:00 and had free access to fresh water throughout the experiment.

### Sample collection and analysis

Cows were milked at 05:00, 13:00, and 21:00 m and milk yield was documented daily. A 50-mL milk sample was taken to determine milk protein (MilkoScan™ FT1, FOSS, Denmark) and somatic cell count (SCC) (Fossomatic 5000, FOSS).

Samples of rumen fluid were taken from each cow at 1-h intervals starting at 04:00 on the sampling day of the 18th week. Collected samples were filtered through 2 layers of gauze and stored at −20°C for LPS analysis.

Blood samples were collected 4 h after feeding on the sampling day of the 18th week. The samples were obtained *via* the lacteal artery and vein in 5-mL vacuum tubes containing sodium heparin. Plasma was isolated from blood samples by centrifugation at 1,469 × *g* at 4°C for 15 min and was stored at −20°C for LPS analysis.

Tissue samples of mammary glands were taken by biopsy on the sampling day from the same quarter of the mammary gland, snap-frozen in liquid nitrogen and then stored at −80°C for further analysis.

### Measurement of rumen pH, LPS and primary pro-inflammatory cytokines

Rumen pH was measured immediately with a pH meter. The concentration of LPS in rumen fluid (CE64406) and plasma (CE80545) was determined by a chromogenic endpoint assay (Chinese Horseshoe Crab Reagent Manufactory Co., Ltd., Xiamen, China) with a minimum detection limit of 0.01 EU/mL. The procedures were performed according to the manufacturer's instructions. The concentrations of the primary pro-inflammatory cytokines IL-1β, IL-6 and TNF-α in the plasma were measured by radioimmunoassay with commercially available human radioimmunoassay kits purchased from the Beijing North Institute of Biological Technology. The detection range of radioimmunoassay kits for IL-1β (cat. C09DJB), IL-6 (cat. C12DJB) and TNF-α (cat. C06PJB).

### Quantitative real-time PCR

Total RNA was extracted from tissue samples of mammary glands using TRIzol reagent (Takara, Dalian, China) according to the manufacturer's protocols. RNA concentration was quantified by measuring the absorbance at 260 nm in a spectrophotometer (Eppendorf Biotechnology, Hamburg, Germany). Then, cDNA was synthesized by reverse transcription using the Oligo (dT) 15-Primer and M-MLV reverse transcriptase (Cat. RR036A, Takara). Primers for target genes were designed using Primer Premier Software 5.0 (Premier Biosoft International, USA) and are presented (Table 2). Quantitative real-time PCR was performed on an ABI 7300 system (Applied Biosystems, Foster City, CA, USA) using a SYBR Premix EX Taqkit (Cat. DRR420A, Takara). PCR amplification was performed using the following protocol: 30 s at 95°C, 5 s at 95°C, and 31 s at annealing temperature 60°C for 40 cycles. The 2^−ΔΔCt^ method was used to analyze qPCR data. Glyceraldehyde-3-phosphate dehydrogenase (GAPDH) was used as an internal reference.

### Signaling pathway analysis

Transcriptome microarray was performed for gene expression profiling of mammary gland tissues. Respective reagent kits from Affymetrix were used for RNA processing, labeling and hybridization. A total of 5μg RNA of each sample was used for cRNA preparation and labeled with the Affymetrix GeneChip Expression 3′ Amplification One-Cycle Target Labeling Kit (Affymetrix, St. Clara, USA). Fragmented cRNA was hybridized for 16 h at 45°C to the GeneChip Bovine Genome (Affymetrix). Microarrays were scanned at 1.56 micron resolution using the GeneChip Scanner 3000 7G (Affymetrix). The RVM *t*-test was applied to filter the differentially expressed genes for the control and experiment groups, as the RVM *t*-test can increase the degrees of freedom effectively when experiments contained small amounts of sample. After the significance analysis and FDR analysis, differentially expressed genes were selected according to the *p*-value threshold. A *P* value < 0.05 was considered statistically significant. Pathway analysis using KEGG, Biocarta and Reatome was used to determine the significant pathways containing the differentially expressed genes identified. Fisher's exact test and the χ^2^ test were used to determine significant pathways, and the threshold of significance was defined by the *P* value and FDR. Enrichment, Re, was calculated according to a previously described equation [48, 49].

### Western blotting

Total protein was extracted from frozen mammary gland tissue with RIPA Lysis Buffer (Cat. SN338, Sunshine Biotechnology Co., Ltd, Nanjing, China) and the protein concentration was determined by BCA assay (Pierce, Rockford, IL, USA). A total of 50 mg of protein extracted from each sample was subjected to electrophoresis by 12% sodium dodecyl sulfate-polyacrylamide gel electrophoresis (SDS-PAGE), and the separated protein was transferred onto a nitrocellulose membrane (Bio Trace, Pall Co., USA). Western blotting analysis for NF-кBp65 (Cat. AN365, Beyotime Biotechnology Co., Ltd, Shanghai, China, 1:500); NF-кBpp65(Cat. AN371, Beyotime Biotechnology Co., Ltd, 1:500); TLR4 (Cat.SC293072, Santa Cruz Biotechnology, Santa Cruz, CA, USA, 1:200); IL-1β (Cat.SC7884, Santa Cruz Biotechnology, 1:200); IL-6 (Cat.SC1265, Santa Cruz Biotechnology, 1:200); TNFα (Cat.SC8301, Santa Cruz Biotechnology, 1:200); αS1-casein (Cat. SC376961, Santa Cruz Biotechnology, 1:200); β-casein (Cat. SC30042, Santa Cruz Biotechnology, 1:200); mTOR (Cat. SC8319, Santa Cruz Biotechnology, 1:200); pmTOR (Cat. SC101738, Santa Cruz Biotechnology, 1:200); p70S6K (Cat. SC9027, Santa Cruz Biotechnology, 1:200) and pp70S6K (Cat. SC7984R, Santa Cruz Biotechnology, 1:200) was performed with primary antibodies and corresponding horseradish peroxidase (HRP)-conjugated secondary antibodies. β-actin (Cat. SC130656, Santa Cruz Biotechnology, 1:500) was used as a reference protein for normalization by western blotting. Finally, the blot was washed and detected by enhanced chemiluminescence (ECL) using a LumiGlo substrate (Super Signal West Pico Trial Kit, Pierce, USA). ECL signals were recorded by an imaging system (Bio-Rad, USA) and analyzed with Quantity One software (Bio-Rad). The protein value was presented as fold change relative to the average value of the LC group's protein.

### Statistical analysis

LPS concentrations in the rumen and in plasma from the lacteal artery and vein, milk protein and SCC were analyzed using a MIXED model with repeated measures using SAS software (SAS Institute, 2004). The expression of target gene mRNA and protein was analyzed using the paired *t*-test. The correlations of mRNA and protein were analyzed by Pearson's test. Differences were considered significant when *P* < 0.05.

## References

[1] Steele MA, Croom J, Kahler M, AlZahal O, Hook SE, Plaizier K, McBride BW (2011). Bovine rumen epithelium undergoes rapid structural adaptations during grain-induced subacute ruminal acidosis. Am J Physiol Regul Integr Comp Physiol.

[2] Cooper RJ, Klopfenstein TJ, Stock RA, Milton CT, Herold DW, Parrott JC (1999). Effects of imposed feed intake variation on acidosis and performance of finishing steers. J Anim Sci.

[3] Gozho GN, Krause DO, Plaizier JC (2007). Ruminal lipopolysaccharide concentration and inflammatory response during grain-induced subacute ruminal acidosis in dairy cows. J Dairy Sci.

[4] Enemark JM (2008). The monitoring, prevention and treatment of sub-acute ruminal acidosis (SARA): a review. Vet J.

[5] Gozho GN, Plaizier JC, Krause DO, Kennedy AD, Wittenberg KM (2005). Subacute ruminal acidosis induces ruminal lipopolysaccharide endotoxin release and triggers an inflammatory response. J Dairy Sci.

[6] Khafipour E, Krause DO, Plaizier JC (2009). A grain-based subacute ruminal acidosis challenge causes translocation of lipopolysaccharide and triggers inflammation. J Dairy Sci.

[7] Dong G, Liu S, Wu Y, Lei C, Zhou J, Zhang S (2011). Diet-induced bacterial immunogens in the gastrointestinal tract of dairy cows: impacts on immunity and metabolism. Acta Vet Scand.

[8] Gozho GN, Krause DO, Plaizier JC (2006). Rumen lipopolysaccharide and inflammation during grain adaptation and subacute ruminal acidosis in steers. J Dairy Sci.

[9] Plaizier JC, Krause DO, Gozho GN, McBride BW (2008). Subacute ruminal acidosis in dairy cows: the physiological causes, incidence and consequences. Vet J.

[10] Gruys E, Toussaint MJ, Niewold TA, Koopmans SJ (2005). Acute phase reaction and acute phase proteins. J Zhejiang Univ Sci B.

[11] Bannerman DD, Paape MJ, Hare WR, Hope JC (2004). Characterization of the bovine innate immune response to intramammary infection with Klebsiella pneumoniae. J Dairy Sci.

[12] Sohn MJ, Hur GM, Byun HS, Kim WG (2008). Cyclo(dehydrohistidyl-l-tryptophyl) inhibits nitric oxide production by preventing the dimerization of inducible nitric oxide synthase. Biochem Pharmacol.

[13] Cohen J (2002). The immunopathogenesis of sepsis. Nature.

[14] Groenen MAM, Poel JJ (1994). Regulation of expression of milk protein genes: a review. Livest Prod Sci.

[15] Wang X, Proud CG (2006). The mTOR pathway in the control of protein synthesis. Physiology (Bethesda).

[16] Yang J, Kennelly JJ, Baracos VE (2000). Physiological levels of Stat5 DNA binding activity and protein in bovine mammary gland. J Anim Sci.

[17] AlZahal O, Rustomo B, Odongo NE, Duffield TF, McBride BW (2007). Technical note: A system for continuous recording of ruminal pH in cattle. J Anim Sci.

[18] Prizant RL, Barash I (2008). Negative effects of the amino acids Lys, His, and Thr on S6K1 phosphorylation in mammary epithelial cells. J Cell Biochem.

[19] Toerien CA, Trout DR, Cant JP (2010). Nutritional stimulation of milk protein yield of cows is associated with changes in phosphorylation of mammary eukaryotic initiation factor 2 and ribosomal s6 kinase 1. J Nutr.

[20] Gunther J, Koczan D, Yang W, Nurnberg G, Repsilber D, Schuberth HJ, Park Z, Maqbool N, Molenaar A, Seyfert HM (2009). Assessment of the immune capacity of mammary epithelial cells: comparison with mammary tissue after challenge with Escherichia coli. Vet Res.

[21] Vanselow J, Yang W, Herrmann J, Zerbe H, Schuberth HJ, Petzl W, Tomek W, Seyfert HM (2006). DNA-remethylation around a STAT5-binding enhancer in the alphaS1-casein promoter is associated with abrupt shutdown of alphaS1-casein synthesis during acute mastitis. J Mol Endocrinol.

[22] Gunther J, Esch K, Poschadel N, Petzl W, Zerbe H, Mitterhuemer S, Blum H, Seyfert HM (2011). Comparative kinetics of Escherichia coli- and Staphylococcus aureus-specific activation of key immune pathways in mammary epithelial cells demonstrates that S. aureus elicits a delayed response dominated by interleukin-6 (IL-6) but not by IL-1A or tumor necrosis factor alpha. Infect Immun.

[23] Petzl W, Zerbe H, Gunther J, Yang W, Seyfert HM, Nurnberg G, Schuberth HJ (2008). Escherichia coli, but not Staphylococcus aureus triggers an early increased expression of factors contributing to the innate immune defense in the udder of the cow. Vet Res.

[24] Danielsen M, Codrea MC, Ingvartsen KL, Friggens NC, Bendixen E, Rontved CM (2010). Quantitative milk proteomics—host responses to lipopolysaccharide-mediated inflammation of bovine mammary gland. Proteomics.

[25] Gunther J, Petzl W, Zerbe H, Schuberth HJ, Koczan D, Goetze L, Seyfert HM (2012). Lipopolysaccharide priming enhances expression of effectors of immune defence while decreasing expression of pro-inflammatory cytokines in mammary epithelia cells from cows. BMC Genomics.

[26] Garrett EF, Pereira MN, Nordlund KV, Armentano LE, Goodger WJ, Oetzel GR (1999). Diagnostic methods for the detection of subacute ruminal acidosis in dairy cows. J Dairy Sci.

[27] Oba M, Allen MS (2000). Effects of brown midrib 3 mutation in corn silage on productivity of dairy cows fed two concentrations of dietary neutral detergent fiber: 3. Digestibility and microbial efficiency. J Dairy Sci.

[28] Bruckmaier RM (2005). Gene expression of factors related to the immune reaction in response to intramammary Escherichia coli lipopolysaccharide challenge. Journal of Dairy Research.

[29] Fujihara M, Muroi M, Tanamoto K, Suzuki T, Azuma H, Ikeda H (2003). Molecular mechanisms of macrophage activation and deactivation by lipopolysaccharide: roles of the receptor complex. Pharmacol Ther.

[30] Medzhitov R, Preston-Hurlburt P, Kopp E, Stadlen A, Chen C, Ghosh S, Janeway CA (1998). MyD88 is an adaptor protein in the hToll/IL-1 receptor family signaling pathways. Mol Cell.

[31] Wang J, Lin D, Peng H, Shao J, Gu J (2014). Cancer-derived immunoglobulin G promotes LPS-induced proinflammatory cytokine production *via* binding to TLR4 in cervical cancer cells. Oncotarget.

[32] Kimura A, Naka T, Muta T, Takeuchi O, Akira S, Kawase I, Kishimoto T (2005). Suppressor of cytokine signaling-1 selectively inhibits LPS-induced IL-6 production by regulating JAK-STAT. Proc Natl Acad Sci U S A.

[33] Kawai T, Akira S (2007). Signaling to NF-kappaB by Toll-like receptors. Trends Mol Med.

[34] Yamamoto M, Sato S, Hemmi H, Hoshino K, Kaisho T, Sanjo H, Takeuchi O, Sugiyama M, Okabe M, Takeda K, Akira S (2003). Role of adaptor TRIF in the MyD88-independent toll-like receptor signaling pathway. Science.

[35] Chang G, Zhuang S, Seyfert HM, Zhang K, Xu T, Jin D, Guo J, Shen X (2015). Hepatic TLR4 signaling is activated by LPS from digestive tract during SARA, and epigenetic mechanisms contribute to enforced TLR4 expression. Oncotarget.

[36] Mohapatra DK, Reddy DS, Ramaiah MJ, Ghosh S, Pothula V, Lunavath S, Thomas S, Valli SN, Bhadra MP, Yadav JS (2014). Rugulactone derivatives act as inhibitors of NF-kappaB activation and modulates the transcription of NF-kappaB dependent genes in MDA-MB-231cells. Bioorg Med Chem Lett.

[37] Barash I (2006). Stat5 in the mammary gland: controlling normal development and cancer. J Cell Physiol.

[38] Wheeler TT, Broadhurst MK, Sadowski HB, Farr VC, Prosser CG (2001). Stat5 phosphorylation status and DNA-binding activity in the bovine and murine mammary glands. Mol Cell Endocrinol.

[39] Bhaskar PT, Hay N (2007). The two TORCs and Akt. Dev Cell.

[40] Jeong YJ, Cho HJ, Magae J, Lee IK, Park KG, Chang YC (2013). Ascofuranone suppresses EGF-induced HIF-1alpha protein synthesis by inhibition of the Akt/mTOR/p70S6K pathway in MDA-MB-231 breast cancer cells. Toxicol Appl Pharmacol.

[41] Gingras AC, Gygi SP, Raught B, Polakiewicz RD, Abraham RT, Hoekstra MF, Aebersold R, Sonenberg N (1999). Regulation of 4E-BP1 phosphorylation: a novel two-step mechanism. Genes Dev.

[42] Turnquist HR, Cardinal J, Macedo C, Rosborough BR, Sumpter TL, Geller DA, Metes D, Thomson AW (2010). mTOR and GSK-3 shape the CD4+ T-cell stimulatory and differentiation capacity of myeloid DCs after exposure to LPS. Blood.

[43] Schmitz S, Pfaffl MW, Meyer HH, Bruckmaier RM (2004). Short-term changes of mRNA expression of various inflammatory factors and milk proteins in mammary tissue during LPS-induced mastitis. Domest Anim Endocrinol.

[44] Sriskandan S, Altmann DM (2008). The immunology of sepsis. J Pathol.

[45] Tian B, Nowak DE, Jamaluddin M, Wang S, Brasier AR (2005). Identification of direct genomic targets downstream of the nuclear factor-kappaB transcription factor mediating tumor necrosis factor signaling. J Biol Chem.

[46] Wolfs TG, Buurman WA, Schadewijk A, de Vries B, Daemen MA, Hiemstra PS, van ‘t Veer C (2002). *In vivo* expression of Toll-like receptor 2 and 4 by renal epithelial cells: IFN-gamma and TNF-alpha mediated up-regulation during inflammation. J Immunol.

[47] Bauchart TC, Cui L, Wu G, Burrin DG (2010). Arginine-induced stimulation of protein synthesis and survival in IPEC-J2 cells is mediated by mTOR but not nitric oxide. Am J Physiol Endocrinol Metab.

[48] Kanehisa M, Goto S, Kawashima S, Okuno Y, Hattori M (2004). The KEGG resource for deciphering the genome. Nucleic Acids Res.

[49] Yi M, Horton JD, Cohen JC, Hobbs HH, Stephens RM (2006). WholePathwayScope: a comprehensive pathway-based analysis tool for high-throughput data. BMC Bioinformatics.

